# Supplementary material for: The nuclear receptor ERR cooperates with the cardiogenic factor GATA4 to orchestrate cardiomyocyte maturation
Source: Nat Commun. 2022 Apr 13;13:1991. doi: 10.1038/s41467-022-29733-3 (PMC9008061; doi:10.1038/s41467-022-29733-3)
Supplement: Supplementary file 1 — Supplementary Information [file 41467_2022_29733_MOESM1_ESM.pdf]

Supplementary Information

The Nuclear Receptor ERR Cooperates with the Cardiogenic Factor GATA4 to Orchestrate Cardiomyocyte Maturation

Tomoya Sakamoto, Kirill Batmanov, Shibiao Wan, Yuanjun Guo, Ling Lai, Rick B. Vega, and Daniel P. Kelly

Supplementary Figures 1-8
Supplementary Tables 1-5

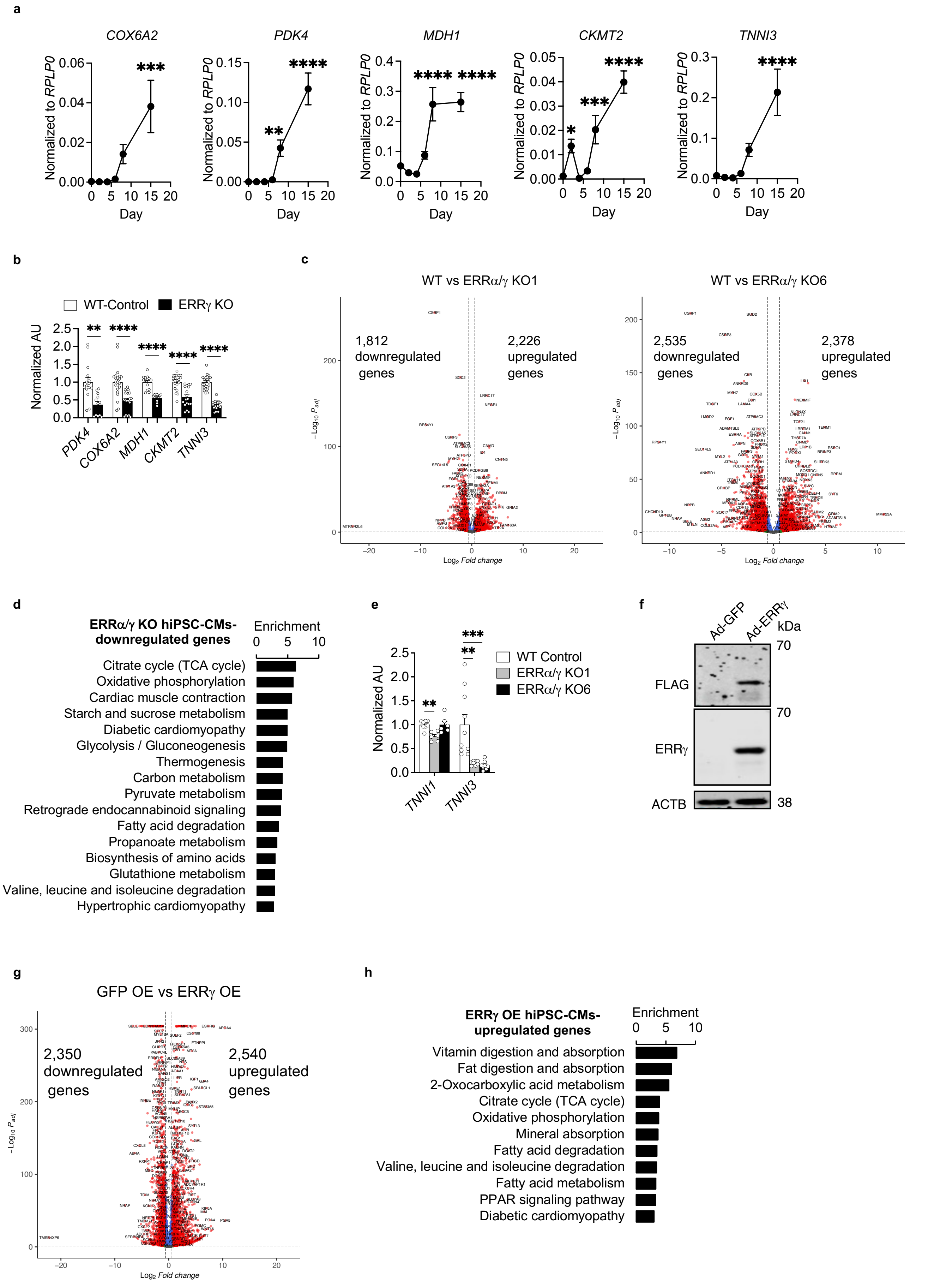
a
COX6A2
PDK4
MDH1
CKMT2
TNNI3
Normalized to RPLP0
Day
b
WT-Control
ERRγ KO
Normalized AU
PDK4
COX6A2
MDH1
CKMT2
TNNI3
c
WT vs ERRα/γ KO1
1,812 downregulated genes
2,226 upregulated genes
WT vs ERRα/γ KO6
2,535 downregulated genes
2,378 upregulated genes
−Log10 P adj
Log2 Fold change
d
ERRα/γ KO hiPSC-CMs-downregulated genes
Enrichment
Citrate cycle (TCA cycle)
Oxidative phosphorylation
Cardiac muscle contraction
Starch and sucrose metabolism
Diabetic cardiomyopathy
Glycolysis / Gluconeogenesis
Thermogenesis
Carbon metabolism
Pyruvate metabolism
Retrograde endocannabinoid signaling
Fatty acid degradation
Propanoate metabolism
Biosynthesis of amino acids
Glutathione metabolism
Valine, leucine and isoleucine degradation
Hypertrophic cardiomyopathy
e
WT Control
ERRα/γ KO1
ERRα/γ KO6
Normalized AU
TNNI1
TNNI3
f
Ad-GFP
Ad-ERRγ
kDa
70
FLAG
70
ERRγ
ACTB
38
g
GFP OE vs ERRγ OE
2,350 downregulated genes
2,540 upregulated genes
−Log10 P adj
Log2 Fold change
h
ERRγ OE hiPSC-CMs-upregulated genes
Enrichment
Vitamin digestion and absorption
Fat digestion and absorption
2-Oxocarboxylic acid metabolism
Citrate cycle (TCA cycle)
Oxidative phosphorylation
Mineral absorption
Fatty acid degradation
Valine, leucine and isoleucine degradation
Fatty acid metabolism
PPAR signaling pathway
Diabetic cardiomyopathy

Supplementary Figure 1. ERR signaling is an activator for both cardiac energy metabolic and structural pathways in hiPSC-CMs.
(a) Real-time quantitative polymerase chain reaction (RT-qPCR)-determined levels of the designated mRNAs during human induced pluripotent stem cell-derived cardiomyocyte (hiPSC-CM) differentiation. Levels of indicated genes are shown as arbitrary units (AU) normalized to *RPLP0* levels at each timepoint. *p<0.05, **p<0.01, ***p<0.001, ****p<0.0001 vs Day 0; one-way ANOVA followed by Dunnett's multiple comparison test. Day 0 and 2, n=7; Day 4, 6, 8, and 15, n=6. (b) The mRNA levels of indicated genes determined by RT-qPCR in wild-type (WT)-Control and ERRγ knockout (KO) hiPSC-CMs. **p<0.01 or ****p<0.0001 , WT-Control vs ERRγ KO, two-tailed student's t-test. *PDK4* (WT-Control, n=15 and ERRγ KO, n=12); *COX6A2* (WT-Control, n=22 and ERRγ KO, n=19); *MDH1* (WT-Control, n=14 and ERRγ KO, n=11); *CKMT2* (WT-Control, n=21 and ERRγ KO, n=18); *TNNI3* (WT-Control, n=22 and ERRγ KO, n=19). (c) Volcano plot of RNA-sequencing (RNA-seq) data showing regulated genes (red) in each ERRα/γ KO hiPSC-CM line compared to wild-type control cells (|Fold change|>1.5 and Benjamini-Hochberg false discovery rate <0.05). (d) Bars represent enrichment score of Kyoto Encyclopedia of Genes and Genomes (KEGG) pathway terms using transcripts downregulated in ERRα/γ KO hiPSC-CMs compared to WT-control. (e) Bar graphs represent mRNA expression levels of *TNNI1* (WT, n=8; ERRα/γ KO1 or 6, n=6) and *TNNI3* (WT, n=10; ERRα/γ KO1 or 6, n=8) by RT-qPCR. **p<0.01, ***p<0.001, one-way ANOVA followed by Dunnett's multiple comparison test. (f) Representative immunoblot images of FLAG, ERRγ, and ACTB levels in hiPSC-CMs following the infection of adenovirus expressing GFP or ERRγ (Ad-GFP or Ad-ERRγ). ACTB was used as a loading control. FLAG expression indicates the levels of overexpressed FLAG-tagged ERRγ. The protein levels were assessed 48 hours after the adenoviral infection. All graphs in a, b and e represent the means +/- SEM. n denotes independent biological replicates. (g) Volcano plot of RNA-seq data showing regulated genes (red) in ERRγ overexpression (OE) hiPSC-CMs compared to GFP OE hiPSC-CMs (|Fold change|>1.5 and adjusted p-values <0.05). (h) Bars represent enrichment score of KEGG pathway terms using transcripts upregulated in ERRγ OE hiPSC-CMs compared to GFP control.

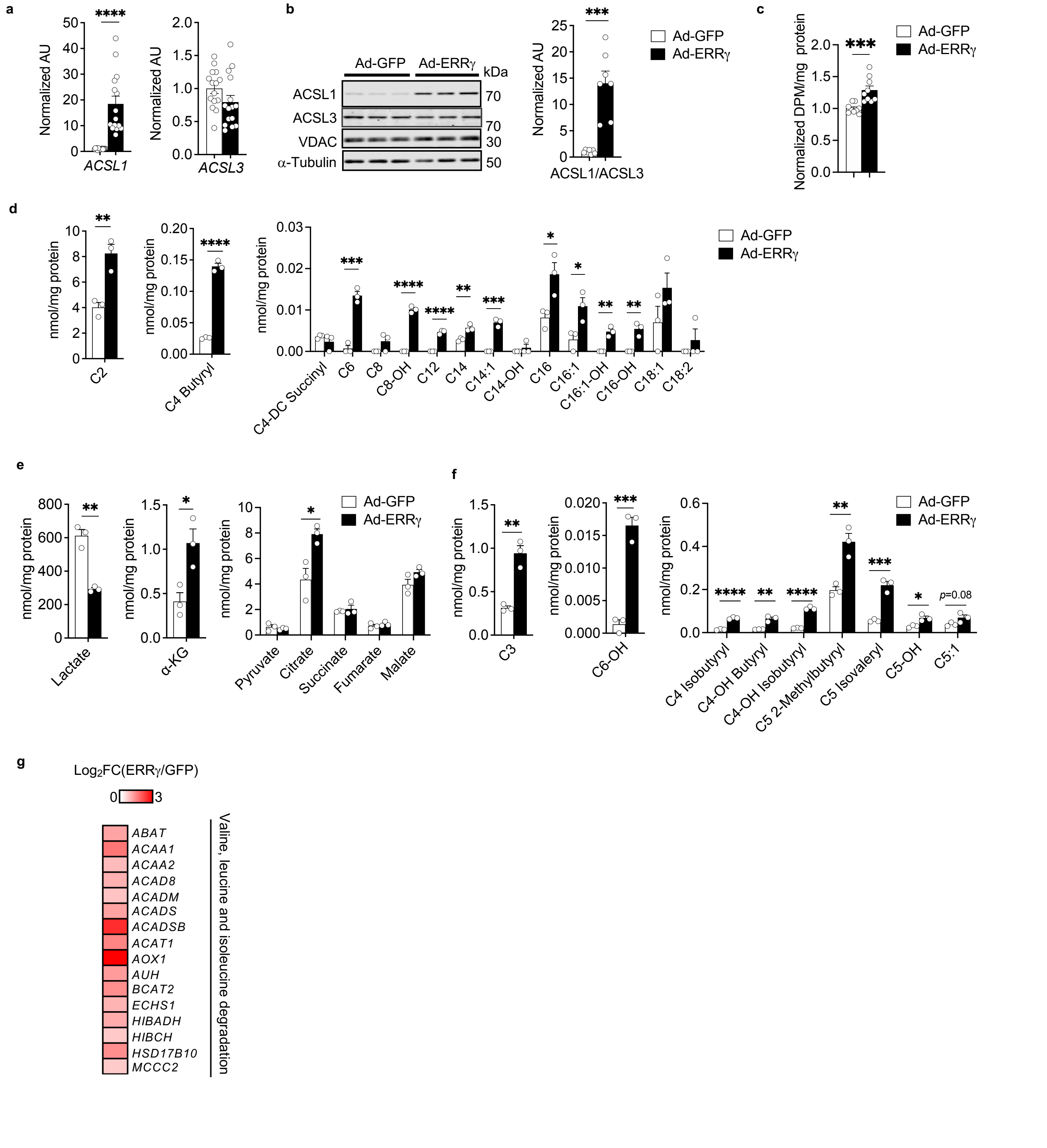

Supplementary Figure 2. Forced expression of ERRγ drives mitochondrial oxidative flux in hiPSC-CMs.
(a) The mRNA levels of *ACSL1* and *ACSL3* determined by real-time quantitative polymerase chain reaction (RT-qPCR) in human induced pluripotent stem cell-derived cardiomyocytes (hiPSC-CMs) following the infection of adenovirus expressing GFP or ERRγ (Ad-GFP; n=15 or Ad-ERRγ; n=15). (b) Representative immunoblot images of ACSL1, ACSL3, VDAC and α-Tubulin levels. VDAC was used as an internal control for mitochondrial content and α-Tubulin was used as a loading control. Bar graphs represents the ratio of ACSL1/3 protein expression normalized to Ad-GFP; n=7. (c) ^{3}H-Palmitate oxidation rates measured in Ad-ERRγ or Ad-GFP. Bars represent mean disintegrations per minute (DPM) normalized to that of Ad-GFP control; n=3. (d) Levels of FAO-derived acylcarnitine species isolated from hiPSC-CMs following Ad-GFP (n=3) or Ad-ERRγ (n=3) were measured by targeted, quantitative metabolomics. (e) Levels of organic acids isolated from hiPSC-CMs following infection by Ad-GFP (n=3) or Ad-ERRγ (n=3) were measured by targeted, quantitative metabolomics. α-KG denotes α-Ketoglutarate. (f) Levels of branched-chain amino acid (BCAA)-catabolism-derived acylcarnitine species isolated from hiPSC-CMs following Ad-GFP (n=3) or Ad-ERRγ (n=3) were measured by targeted, quantitative metabolomics. All data represent the mean +/- SEM. n denotes independent biological replicates. *p<0.05, **p<0.01, ***p<0.001, ****p<0.0001 vs Ad-GFP, two-tailed student's t-test. (g) Heat map representing log$_2$ fold-change (FC) of mRNA levels of genes involved in BCAA catabolism ["Valine, leucine and isoleucine degradation" in Kyoto Encyclopedia of Genes and Genomes (KEGG) pathway] from RNA-sequencing analysis performed in hiPSC-CMs following Ad-ERRγ compared to Ad-GFP.

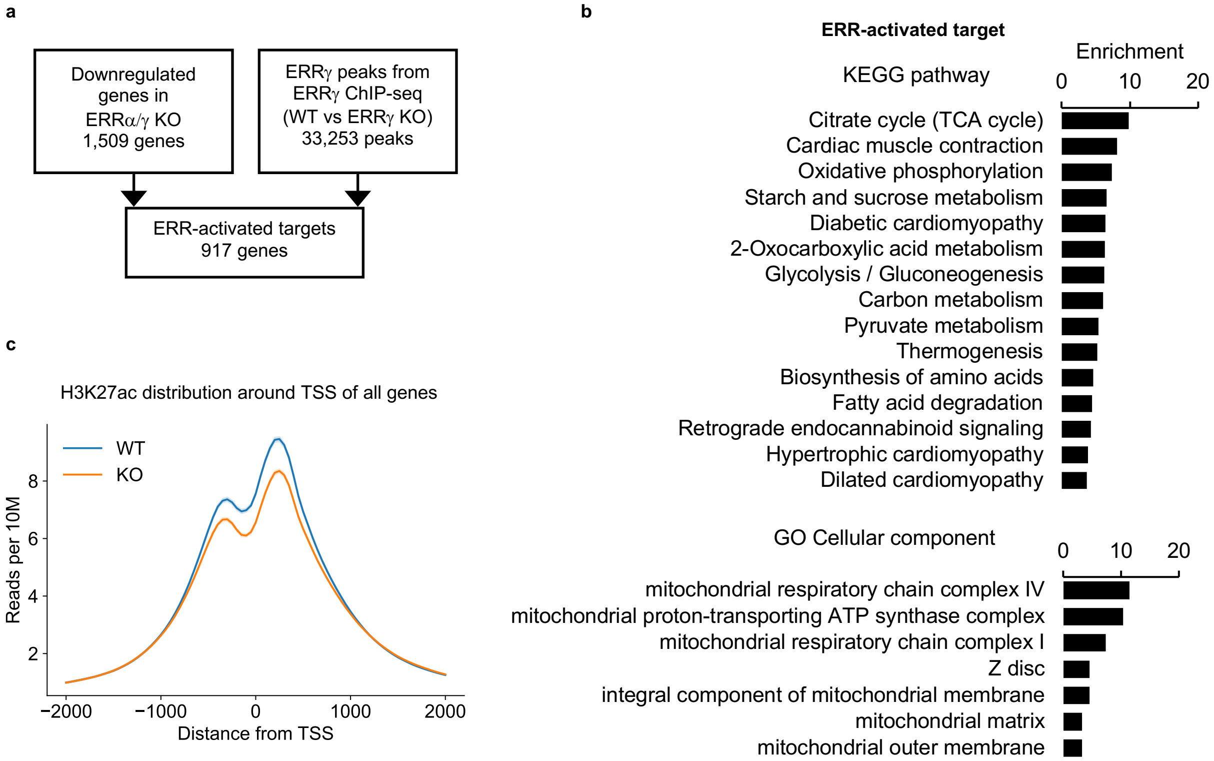

d

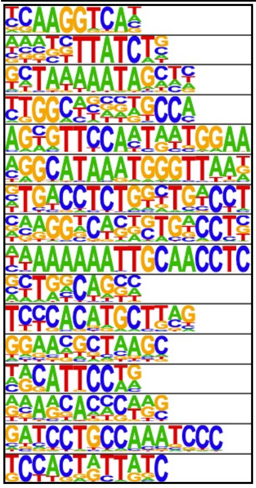

Motif	% of Targets	% of Background	*p*-value
ERR	61.72 %	7.99 %	1e-11452
TRPS1	23.70 %	8.88 %	1e-1204
MEF2	6.20 %	1.60 %	1e-484
NF1	8.65 %	2.90 %	1e-480
ZBTB12	0.76 %	0.01 %	1e-409
ZNF652	0.74 %	0.01 %	1e-397
RAR	0.87 %	0.01 %	1e-367
ESR	5.11 %	1.42 %	1e-362
MAFK	0.69 %	0.01 %	1e-362
MEIS1	41.27%	30.44 %	1e-325
MX11	0.58 %	0.01 %	1e-293
CRX	0.60 %	0.01 %	1e-257
TEAD	7.01 %	3.05 %	1e-244
NKX2-5	0.42 %	0.01 %	1e-161
NFIA	0.45 %	0.01 %	1e-155
GATA	0.40 %	0.01 %	1e-150

e

ERR-activated targets

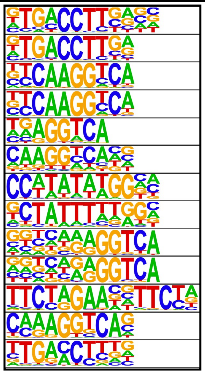

Motif	*p*-value
ERR	1e-9
ERR	1e-7
NR5A2	1e-5
NR5A2	1e-5
THR	1e-3
SF1	1e-3
CArG	1e-3
MEF2	1e-2
COUP-TFII	1e-2
EAR2	1e-2
HRE	1e-2
ERR	1e-2
RAR	1e-2

ERR-suppressed targets

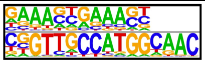

Motif	*p*-value
IRF2	1e-2
RFX	1e-2

Supplementary Figure 3. ERRγ directly activates the transcription of genes coding cardiac metabolic and structural proteins.
(a) Schematic indicates the intersection analysis with RNA-sequencing (RNA-seq) in ERRα/γ knockout (KO) human induced pluripotent stem cell-derived cardiomyocytes (hiPSC-CMs; GSE165963) and published ERRγ chromatin immunoprecipitation sequencing (ChIP-seq) in hiPSC-CMs (GSE113784). (b) Bar graphs represent enrichment score of Kyoto Encyclopedia of Genes and Genomes (KEGG) and Gene Ontology (GO) Cellular Component pathway terms using the ERR-activated genes defined by the intersection analysis. (c) Aggregation plots represent H3K27ac ChIP-seq signals around transcription start sites (TSS) in WT and ERRγ KO hiPSC-CMs. Significant enrichment of H3K27ac ChIP-seq signals were observed in both groups. (d) De novo motif enrichment results within ERRγ peaks in which H3K27ac deposition was not altered by ERRγ KO. (e) Known motif enrichment results are presented for each ERR-activated and ERR-suppressed targets. The *p*-values were calculated with Fisher's exact test in d and e.

a

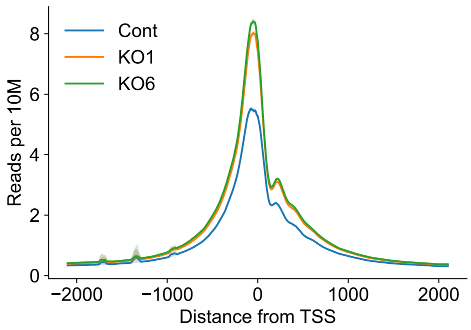
Cont
KO1
KO6
Reads per 10M
Distance from TSS
8
6
4
2
0
−2000
−1000
0
1000
2000

b

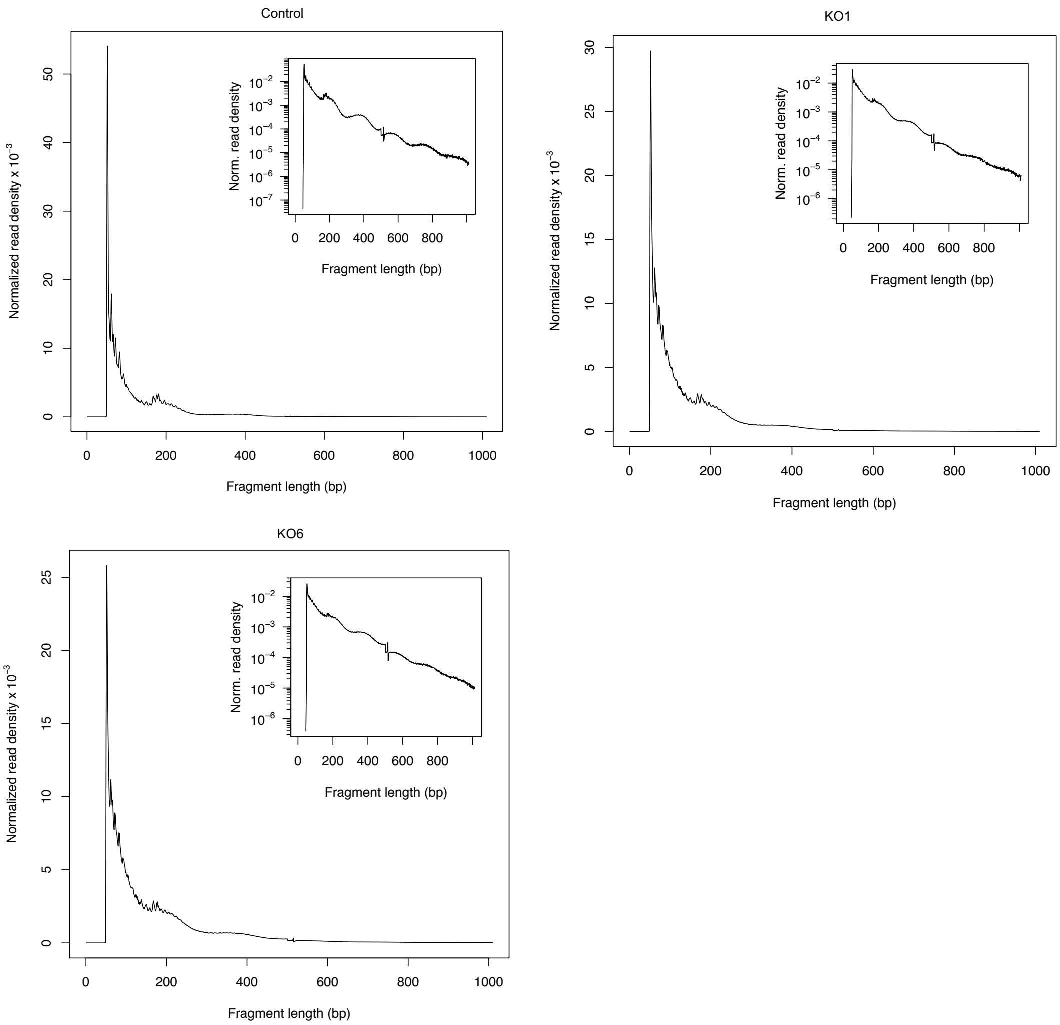
Control
KO1
KO6
Normalized read density x 10^-3
Fragment length (bp)
Norm. read density
0
200
400
600
800
1000

c

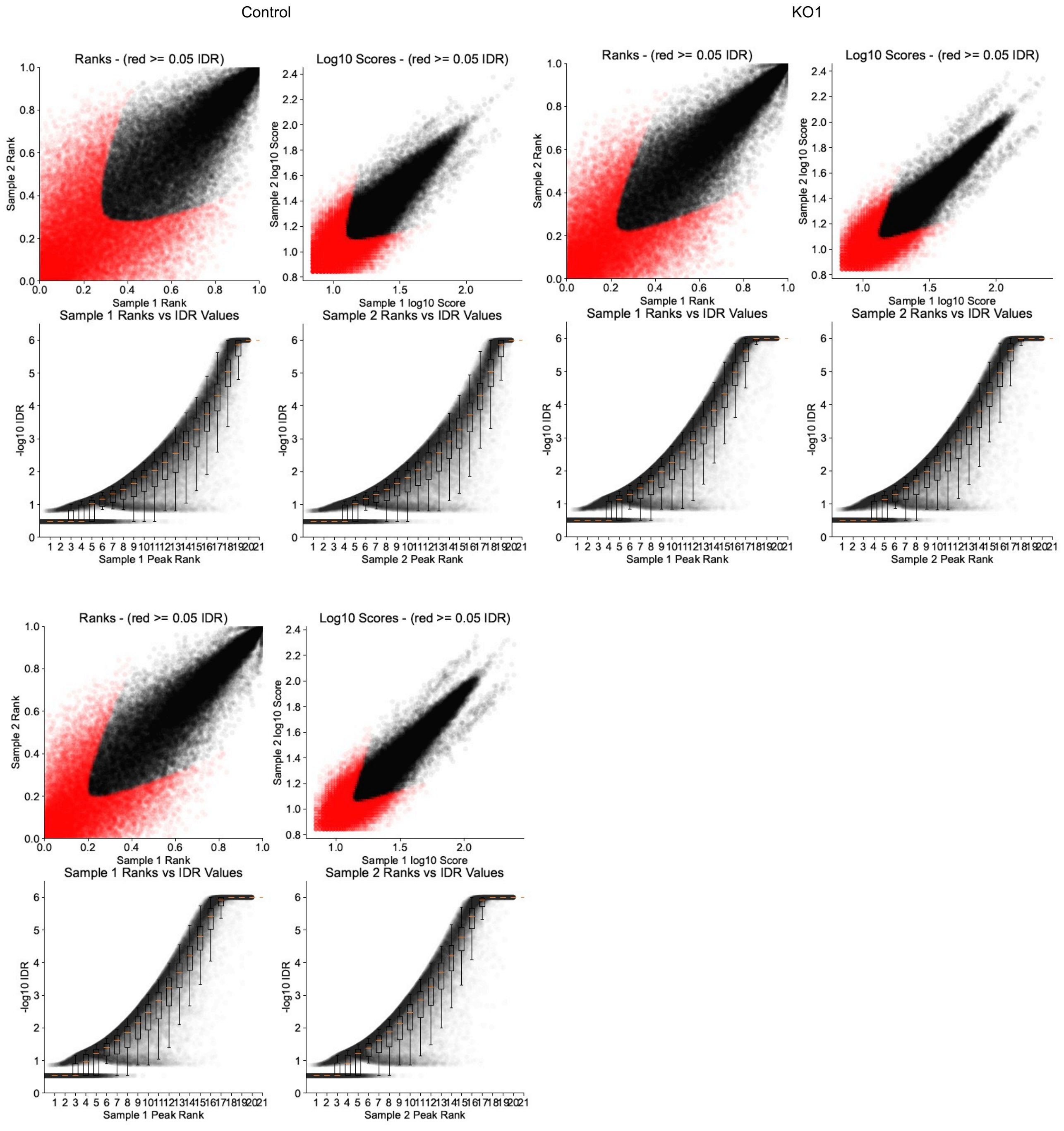

Supplementary Figure 4. Evaluation of the data quality of ATAC-seq conducted on WT and ERRα/γ KO hiPSC-CMs. We evaluated our assay for transposase accessible chromatin with high-throughput sequencing (ATAC-seq) data according to Encyclopedia of DNA Elements (ENCODE) data quality standards for ATAC-Seq (https://www.encodeproject.org/atac-seq/). (a) Transcriptional start site (TSS) enrichment values in ATAC-seq data from wild-type control (Cont) and ERRα/γ KO hiPSC-CMs derived from two distinct lines (KO1 and KO6) are presented. Ideal TSS enrichment is > 10-fold over flanking regions. (b) Representative fragment size distribution of each data. Nucleosome-free region (<100bp) and fragments spanning one or multiple nucleosomes are visible as peaks on the fragment size distribution plot. (c) Replicate concordance was measured by calculating Irreproducible Discovery Rate (IDR) values. The experiment passes the ENCODE criteria if both rescue, and self consistency ratios are less than 2. IDR Self consistency ratio: Cont= 1.01998451, KO1= 1.07301239, and KO6= 1.19442941; IDR Rescue ratio: Cont= 1.272725, KO1= 1.161522, and KO6= 1.145185.

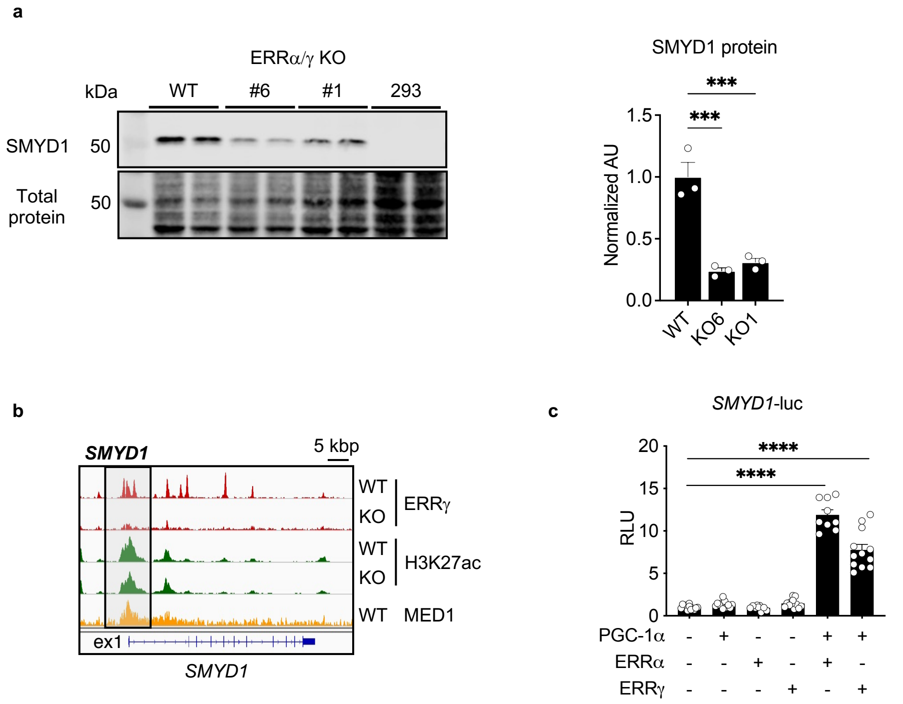

Supplementary Figure 5. ERRα/γ directly regulates the expression level of SMYD1 in hiPSC-CMs.
(a) Representative images of SMYD1 immunoblot. Total protein staining is used as a loading control. Bars represent the quantification of SMYD1 protein expression levels in wild-type control (WT; n=3) and ERRα/γ knockout (KO; n=3) human induced pluripotent stem cell-derived cardiomyocytes (hiPSC-CMs). ***p<0.001, one-way ANOVA, Dunnett's multiple comparison test. Whole cell lysate of AD-293 cells was used as the negative control for SMYD1 protein expression. (b) Genome browser of *SMYD1* locus to show ERRγ (GSE113784), H3K27ac (GSE165965), and MED1 (GSE85631) peaks. (c) The marked region (chr2:88066119-88069666) in (b) was cloned into pGL3 reporter plasmid. Bar graphs represent relative light unit (RLU) derived from *SMYD1*-luc reporter construct. GFP, PGC-1α, ERRγ, and PGC-1α/ERRγ, n=12; ERRα and PGC-1α/ERRα, n=9. One-way ANOVA followed by Dunnett's multiple comparison test was used. ****p<0.0001 compared to GFP transfected group. n denotes independent biological replicates. Data are presented as the means +/- SEM.

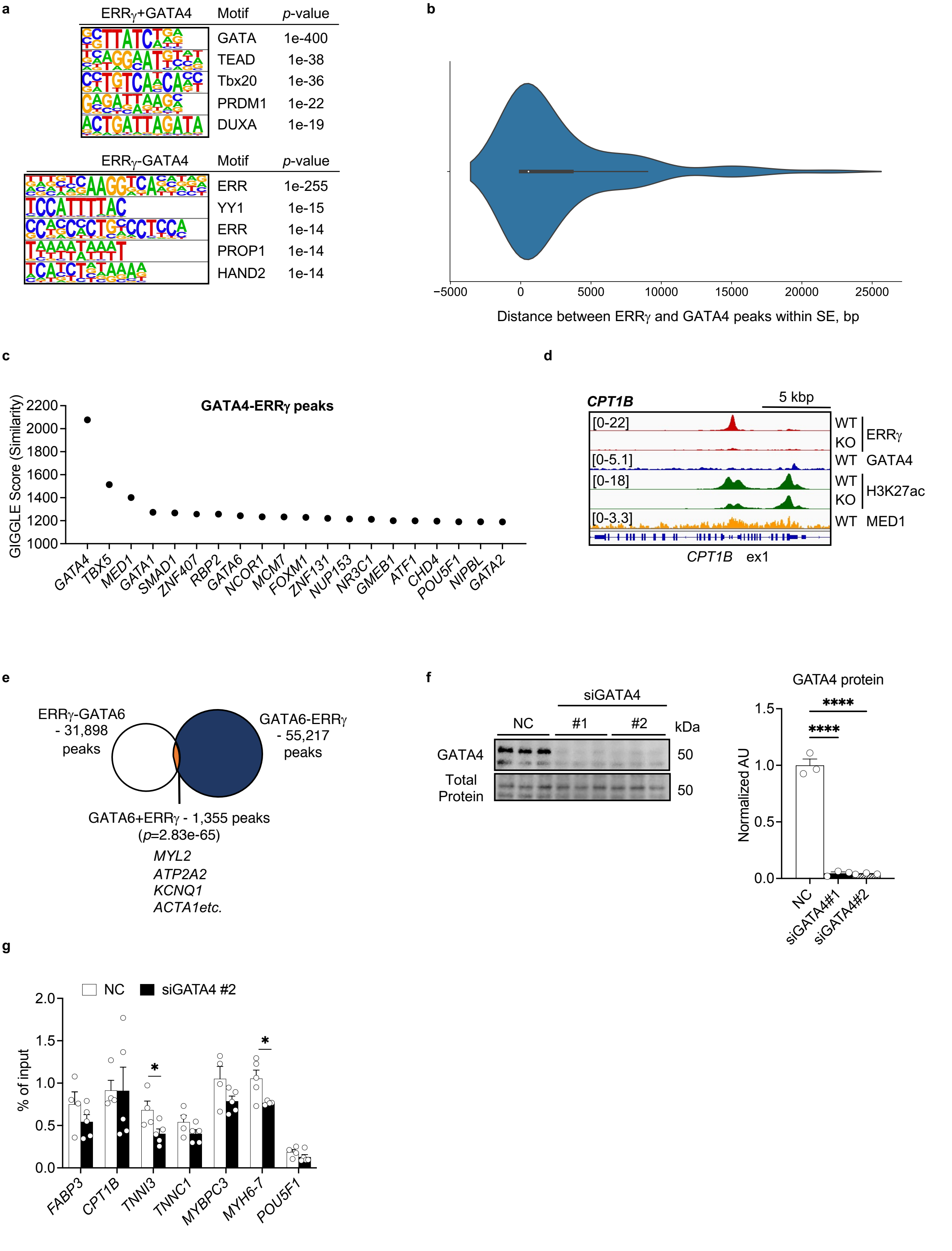

Supplementary Figure 6. GATA4 but not GATA6 often colocalizes with ERRγ on genes coding cardiac-enriched structural proteins.
(a) Differential motif analysis of ERRγ+GATA4 peaks vs. ERRγ–GATA4 peaks (top) and ERRγ–GATA4 peaks vs. ERRγ+GATA4 peaks (bottom). The *p*-values were calculated with Fisher's exact test. (b) The violin plot shows smoothed density (in arbitrary units) of the distribution of distances between ERRγ and GATA4 peaks within a single super-enhancer (SE) in human induced pluripotent stem cell-derived cardiomyocytes (hiPSC-CMs). White dot: median; horizontal black box: 25-75 percentile; whiskers: 1.5 interquartile range; blue area: width proportional to the density of points in the vicinity of the X coordinate. The sample number = 99, independent SEs containing both ERRγ and GATA4 peaks. (c) Top 20 transcription factors significantly overlapped with GATA4 peaks without ERRγ peaks (GATA4-ERRγ) in Cistrome Data Base Toolkit analysis (http://dbtoolkit.cistrome.org/) were plotted. Y axis represents the GIGGLE score which means the similarity between published data sets and given peak set. (d) Genomic browser track of *CPT1B* locus as an ERRγ target. (e) Venn diagram indicates the overlapped peaks from published GATA6 chromatin immunoprecipitation sequencing (ChIP-seq) and ERRγ ChIP-seq (GSE113784) in hiPSC-CMs. GATA6 and ERRγ-shared peaks (GATA6+ERRγ) are highlighted by orange. The *p*-value was calculated with Fisher's exact test. (f) Representative immunoblot image of GATA4 in hiPSC-CMs transfected with negative control (NC) or two distinct siRNAs targeting GATA4 (siGATA4#1 or #2). Total protein staining was used as a loading control. Bar graphs represent the quantification of GATA4 protein amount (n=3). ****$p<0.0001$ vs NC; one-way ANOVA followed by Dunnett's multiple comparison test. (g) Levels of ERRγ occupation on the indicated targets in negative control (NC, n=4) or siGATA4 #2 (n=5)-transfected hiPSC-CMs as measured by ChIP-quantitative polymerase chain reaction. *$p<0.05$ vs WT, two-tailed student's t-test. Stem cell enhancer region on *POU5F1* was used as a negative control. All bars in f and g represent the means ± SEM. n denotes independent biological replicates in f and g.

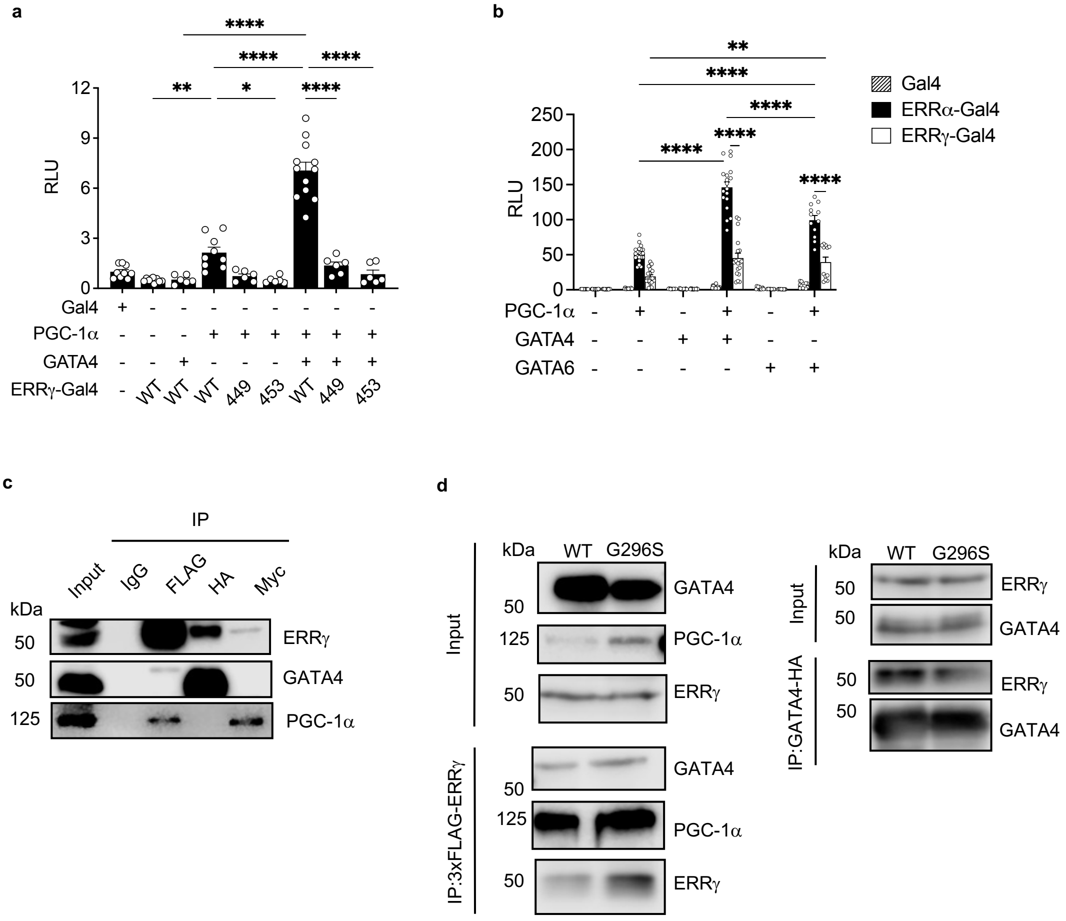

Supplementary Figure 7. The ERRγ-PGC-1α cooperates with GATA4.

(a) A series of ERRγ-Gal4 activation function 2 (AF2) mutant reporter experiments were performed in AD-293 cells with PGC-1α and GATA4. 449 and 453 represent L449A-F450A and M453A-L454A AF2 mutants respectively. Bar graphs represent relative light unit (RLU) from pG5luc construct and the means +/- SEM. Gal4, ERRγ-Gal4, and PGC-1α/ERRγ-Gal4, n=9; PGC-1α/GATA4/ERRγ-Gal4, n=12; PGC-1α/ERRγ L449A-F450A-Gal4, PGC-1α/ERRγ M453A-L454A-Gal4, PGC-1α/GATA4/ ERRγ L449A-F450A-Gal4, and PGC-1α/GATA4/ERRγ M453A-L454A-Gal4, n=6. *p<0.05, **p<0.01, ****p<0.0001, one-way ANOVA followed by Tukey's multiple comparisons test. (b) ERRα or ERRγ-Gal4 system was employed to examine the interaction of GATA4 or 6. Gal4 with or without PGC-1α, n=18; ERRα-Gal4, or ERRγ-Gal4 with or without PGC-1α, n=21; Gal4 and GATA4 with or without PGC-1α, n=15; ERRα-Gal4, or ERRγ-Gal4 and GATA4 with or without PGC-1α, n=18; Gal4, ERRα-Gal4, or ERRγ-Gal4 and GATA6 with or without PGC-1α, n=12. Bars represent RLU and the means +/- SEM. **p<0.01, ****p<0.0001, two-way ANOVA followed by Tukey's multiple comparisons test. n denotes independent biological replicates. (c) Representative immunoblot immunoprecipitation (IP) images to show overexpressed PGC-1α tagged with Myc/His, ERRγ tagged with 3xFLAG, GATA4 tagged with human influenza hemagglutinin (HA) in AD293 cells. (d) Representative immunoblot IP with anti-FLAG or anti-HA images to show overexpressed ERRγ, PGC-1α, and WT or G296S GATA4 in AD293 cells.

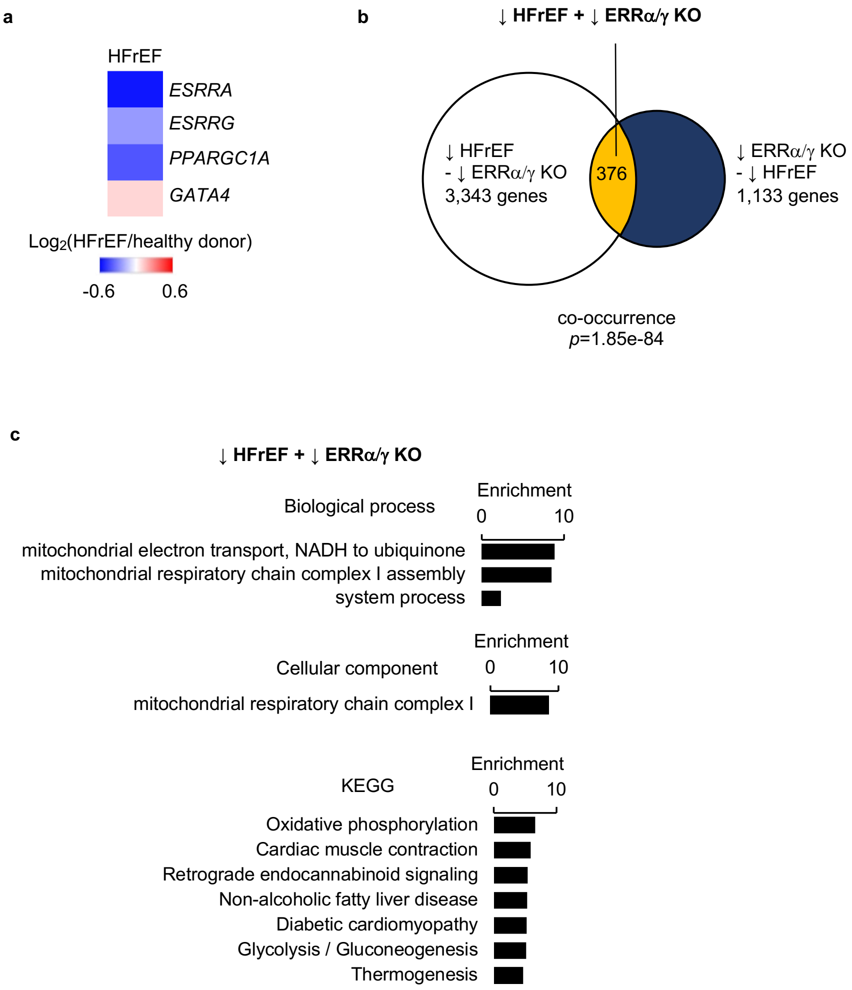

Supplementary Figure 8. Assessment of the PGC-1α/ERR/GATA4 transcriptional circuitry in human heart disease. (a) The heatmap represents Log_2 [heart failure with reduced ejection fraction (HFrEF)/donor control] of the indicated genes in the published RNA-sequencing (RNA-seq) dataset (https://zenodo.org/record/4114617#.YYGfOL3MLUJ) from right ventricle samples of HFrEF and donor control. Changes in *ESRRA*, *ESRRG*, and *PPARGC1A* levels were significant (Benjamini-Hochberg false discovery rate<0.05). (b) Venn diagram shows the intersection of significantly downregulated genes in ERRα/γ KO hiPSC-CMs and HFrEF. The *p*-value was calculated with Fisher's exact test. (c) Bar graphs represent significantly enriched Gene Ontology (GO) Biological process, GO Cellular component, and Kyoto Encyclopedia of Genes and Genomes (KEGG) pathway terms with the commonly downregulated genes (highlighted with yellow in b) in ERRα/γ KO hiPSC-CMs and HFrEF.

Supplementary Table 1. Row-wise z-scores of FPKM values of the annotated-genes associated with both cardiac SE and ERRγ binding sites in hiPSC-CMs.

Gene name	WT_1	WT_2	WT_3	KO1_1	KO1_2	KO6_1	KO6_2	KO6_3
SRSF2	1.491202282	-0.953351718	-0.639713543	1.00264854	-1.739789238	0.522664046	0.384906515	-0.068566885
ZFAND5	1.216273898	-0.596917315	0.814566932	0.182977176	-2.250302801	0.04044577	-0.039728929	0.632685268
MAT2A	2.133536182	-0.447481997	0.093134491	0.210309413	-1.772523267	0.076683037	-0.191788069	-0.101869789
KLF9	1.107925236	0.31611589	0.966167675	0.453406243	0.901625399	-1.279778666	-1.380213996	-1.085247781
STOM	0.273283718	0.643426137	1.631014632	0.724979589	0.291327624	-1.223681419	-1.218273792	-1.122076489
GADD45A	1.273400705	-0.841289086	1.441412201	1.133231071	-0.928995305	-0.651077695	-0.667753252	-0.758928639
CDC42EP3	1.239275613	0.041032701	1.009167961	1.276386677	-0.248927347	-1.304634837	-0.891661797	-1.120638972
MICAL3	2.359127081	-0.207803242	-0.053959728	-1.239560972	0.185660165	-0.748823915	0.179254214	-0.473893603
BOLA1	0.513116465	1.675159504	1.106222275	-1.327505423	0.167422199	-0.214192967	-0.843881576	-1.076340478
SRL	0.870999996	1.509117226	1.069454938	-0.522421626	0.221822941	-1.095984237	-0.718682413	-1.334306826
IRF2BPL	0.687892181	1.519458387	1.134310021	-0.512785928	0.371702952	-1.309837558	-1.061893497	-0.828846557
MYH6	0.862482219	1.18568879	1.571818633	-0.910822398	-0.026975306	-1.099343177	-0.581582689	-1.001266072
IRF2BP2	0.608031832	0.724279667	1.891639316	-1.05551168	0.418841489	-0.803129595	-0.866883675	-0.917267354
ARID1A	0.61387756	1.348007049	1.504784579	-1.415752418	-0.896138469	-0.654252297	-0.551034041	0.050508037
LMOD2	0.539801858	0.753356775	2.175412309	-0.659407689	-0.675256063	-0.712196994	-0.712445144	-0.709265054
FILIP1	0.915069311	-0.162611692	2.20092709	-1.013256186	-0.072282478	-0.838907976	-0.39808751	-0.630850558
LMO7	1.403073967	-0.378876861	1.895570663	-1.193597509	-0.203006629	-0.649500939	-0.324434274	-0.549228417
RND3	1.574556659	0.297921078	1.433726039	0.09807875	-0.257730335	-1.053811924	-1.053411173	-1.039329094
NUAK1	1.557838073	0.188278693	1.532252503	0.089627761	-0.336562716	-0.996836411	-0.966755905	-1.067841998
SAMD4A	1.230119768	0.000202847	1.964850592	-0.139988311	-0.317107571	-0.969514541	-0.917052741	-0.851510041
SCHIP1	1.369411032	0.184033356	1.86679998	-0.292782268	-0.549191486	-0.864455909	-0.820125581	-0.893689124
ADAM19	1.495209005	-0.061353317	1.857250837	-0.448305863	-0.451877076	-0.780852953	-0.796697599	-0.813373034
PFDN1	1.493100118	0.454089059	1.676608134	-0.555742697	-0.511619823	-0.892924702	-0.801029595	-0.862480495
JPH2	1.632500457	0.461289047	1.549835449	-0.6730146	-0.547173172	-0.846401308	-0.725663432	-0.851372441
SYNPO2L	1.563121787	0.413175893	1.655760694	-0.674557676	-0.697828932	-0.765339286	-0.7387934	-0.755539078
CENPN	1.275809697	0.635016447	1.776439411	-0.582404504	-0.813983967	-0.560053342	-0.861759296	-0.869064447
XIRP1	1.295210934	0.531267896	1.824570385	-0.563376534	-0.696013461	-0.826029308	-0.756592988	-0.809036925
MYL12A	1.420611797	0.605528282	1.675475412	-0.550771448	-0.659814788	-0.811374552	-0.827052387	-0.852602316
CMYA5	1.152996947	0.572065475	1.912559662	-0.745176941	-0.733530154	-0.813471402	-0.785092981	-0.560350607
DAPK3	1.070126881	0.382551823	2.036100931	-0.422603636	-0.613176594	-0.781368809	-0.82025009	-0.851380506
TTN	1.589671336	0.337689485	1.624152096	-1.067573451	-0.50948733	-0.779257874	-0.586131655	-0.609062606
SLMAP	1.43009841	0.12572858	1.887001416	-0.762593828	-0.676068054	-0.648565064	-0.669001368	-0.686600091
ANKRD1	1.527422151	0.048875468	1.828541968	-0.645978102	-0.663659282	-0.697755107	-0.70018976	-0.697257336
SLC38A1	2.335401895	0.134654624	0.709989419	-0.624808372	-0.66315269	-0.619418753	-0.637919238	-0.634746885
TNNC1	2.022070763	0.429769467	1.093215423	-0.785563786	-0.721364274	-0.658344016	-0.624267903	-0.755515674
PALM2AKAP2	2.062648084	0.359585331	1.07269444	-0.817635865	-0.721035827	-0.668045714	-0.621097828	-0.667112622
FSD2	2.170370078	0.360572174	0.870083356	-0.661872008	-0.915304547	-0.501437263	-0.641794307	-0.680617483
MYL3	2.136870611	0.42519886	0.910051452	-0.701191802	-0.791647695	-0.645055565	-0.678164031	-0.65606183
FLNC	2.180073608	0.278654702	0.901040657	-0.432889795	-0.634672617	-0.82862841	-0.661440238	-0.802137907
NAV1	2.177045813	0.395221444	0.843878735	-0.734414266	-0.493255307	-0.74986166	-0.640642314	-0.797972446
SH3BGR	2.052903969	0.549648783	0.920546799	-0.550913467	-0.45020214	-0.809971279	-0.791468251	-0.920544416
RAPGEF2	1.937012825	0.019546535	1.40493295	-0.58449645	-0.737619749	-0.671244597	-0.655937184	-0.712194329
SLC20A2	1.859591816	0.270413428	1.402057248	-0.676707128	-0.678700628	-0.731113401	-0.672067767	-0.773473569
ACTC1	1.902715233	0.334410468	1.315440959	-0.673114569	-0.706497873	-0.754286216	-0.638694597	-0.779973404
BMPR1A	1.972000705	-0.160788587	1.373192627	-0.369074386	-0.484565086	-0.857640235	-0.800153285	-0.672971754
RBM20	2.125255227	-0.006098742	1.123901248	-0.509874264	-0.508015842	-0.863885291	-0.559801292	-0.801481044
MYLK3	1.451488215	1.088989163	1.283803539	-0.816482067	-0.458030992	-0.833395572	-0.771058104	-0.945314183
PGM1	1.490191364	1.066501022	1.286537427	-0.679655442	-0.676729127	-0.806571869	-0.792098163	-0.888175211
HSPB7	1.472033998	1.105751346	1.261110611	-0.65521292	-0.612708162	-0.870090278	-0.773782842	-0.927101752
ANKMY2	1.250648294	1.116279916	1.480024788	-0.757509686	-0.605541061	-0.805976912	-0.866423689	-0.811501651
WIPI1	1.125500789	1.278035395	1.436842702	-0.563039345	-0.721594965	-0.910403371	-0.909951235	-0.735389969
PRKAR1A	1.636689294	0.582308291	1.476908419	-0.821313401	-0.920103708	-0.619053064	-0.618529152	-0.71690668
LRRFIP1	1.652374203	0.85726383	1.279880152	-0.768585822	-0.757099669	-0.880927129	-0.707175825	-0.675729741
FABP3	1.543053395	0.997291731	1.293991981	-0.736681151	-0.853650616	-0.743011983	-0.749022425	-0.751970932
SMYD1	1.667242691	0.728340825	1.354984133	-0.741035574	-0.66638837	-0.827787285	-0.640956444	-0.874399976
ACTN2	1.625679054	0.708734579	1.423277019	-0.723887326	-0.765492728	-0.775944549	-0.704437919	-0.787928129
MXRA7	1.685627337	0.658502829	1.388639743	-0.811878335	-0.736258611	-0.769595608	-0.675516842	-0.739520513
TRIM55	1.483444237	0.900044617	1.432716472	-0.871498972	-0.716183413	-0.703585448	-0.705646247	-0.819291246
MYH7	1.508001439	0.886248528	1.422285926	-0.765756123	-0.710033647	-0.784489838	-0.759735035	-0.796521252
CSRP3	1.526730063	0.749481546	1.500545173	-0.717586581	-0.735779651	-0.779769095	-0.769436313	-0.774185142
BZW2	1.411986151	0.848666119	1.54456296	-0.796466659	-0.791250639	-0.756172659	-0.716229794	-0.745095478
STK39	1.903997807	0.56940156	1.166681416	-0.776456562	-0.876865254	-0.642318677	-0.697892029	-0.646548261
ROR1	1.796808273	0.719836524	1.20777368	-0.791706934	-0.746041495	-0.680168647	-0.83637032	-0.670131082
PYGB	1.929536061	0.553157024	1.140763301	-0.698047314	-0.637175711	-0.790683828	-0.626717751	-0.870831782
GPAT3	1.94826303	0.709052908	0.977185674	-0.62726881	-0.614693129	-0.937351451	-0.589943517	-0.865244705
AFG3L2	1.767035865	0.841692828	1.141317097	-0.624291919	-0.82987182	-0.692821052	-0.693823046	-0.909237953
POPDC2	1.786561391	0.547154204	1.318497804	-0.552075347	-0.572687226	-0.873887553	-0.76842428	-0.885138993
CMC2	1.752104804	0.770394372	1.222196805	-0.640737701	-0.645112855	-0.842511155	-0.803290821	-0.813043448
PLN	1.751818882	0.763644097	1.226718679	-0.694968877	-0.59171085	-0.8302193	-0.81296442	-0.812318211
KIF13A	1.700975066	0.920749619	1.127676004	-1.067305019	-0.53467815	-0.843944092	-0.667016622	-0.636456804
ST6GAL1	1.832808644	0.927188865	0.956571612	-1.025699509	-0.635095246	-0.717974387	-0.682259784	-0.655540195
TNNT2	1.774003463	0.988629439	0.944097084	-0.653909518	-0.428772244	-0.88185312	-0.674280213	-1.067914891
SORBS2	1.672428636	0.918235661	1.168377431	-0.861873909	-0.4294365	-0.901265091	-0.664299151	-0.902167078
LDB3	1.884399987	0.557701222	1.105640897	-0.624117928	-0.233529323	-0.951605884	-0.703746153	-1.03474282
AMOTL2	1.436350081	0.889218453	1.361658977	-0.394764629	-0.265880483	-1.084061345	-0.928486405	-1.014034649
JMJD6	1.525391834	0.083925586	1.649779577	-0.856868153	-1.224605009	-0.790195308	-0.119471153	-0.267957375
MYL4	2.218626241	-0.102559277	0.794593982	-0.624140929	-1.27274592	-0.329379899	-0.13765285	-0.546741348
RBM24	1.944844071	-0.301548081	1.060443068	-1.319064432	-1.032881607	-0.162688366	0.180209039	-0.369313693
MTHFR	0.629434037	2.126171467	-0.169494477	0.480728401	-0.179794522	-1.007287918	-0.870303623	-1.009453366
NKX2-5	0.55088394	2.06638068	0.488595174	-0.266852288	-0.256362925	-1.08931909	-0.134570496	-1.358754994
H4C9	-0.485872288	1.755198789	-1.032061077	-0.84985292	1.374649801	-0.933655639	-0.158854327	0.330447661
TXNIP	-0.432735507	1.618472939	0.247124811	-0.80071021	1.656729102	-0.763301332	-0.763384972	-0.76219483
POLR3GL	-0.181084673	1.948338524	0.698642964	-1.296131431	0.733386353	-0.92143719	-0.231682726	-0.750031822
FOS	-0.006523142	-0.39518158	-0.063246404	-0.280309411	2.576472172	-0.553280931	-0.626053935	-0.65187677
FOXP1	-0.130673033	-1.327724128	-0.443536819	-0.900770525	2.208388419	0.423267936	0.351227728	-0.180179578
JOSD2	-1.54402828	0.042657651	0.109471474	1.912156006	0.570125969	-0.293015787	0.3813913	-1.178758333
JPH1	0.210565949	-1.150364224	0.071404372	2.418528463	-0.369889072	-0.697101453	-0.102894001	-0.380250034
CHD2	1.25709636	-1.542494265	-0.437123539	-0.522713214	-0.409014492	-0.198526993	0.017262516	1.835513627
GATA6	-0.781003846	-1.907361408	-0.925042933	0.447732946	0.735538982	0.523103072	1.130708622	0.776324564
FGFR1	-0.385551745	-2.018877414	-0.765486405	1.375322432	0.131289861	1.043230283	0.319806465	0.300266523
ULK4	-0.905035839	-1.435405708	-0.287693826	-0.561005263	-0.227012502	0.419425586	1.446472388	1.550255164
NXF1	0.173318411	-1.499340443	-1.159637904	0.701078667	-1.03285159	1.225809214	0.638261756	0.953361889
OPTC	-0.818403193	-0.818403193	-0.818403193	0.307470831	-0.818403193	1.069186471	-0.12015382	2.017109291

Supplementary Table 1 shows the row-wise z-scores of fragments per kilobase of exon per million reads mapped (FPKM) values of the annotated-genes associated with both cardiac super-enhancer regions (SEs) defined by MED1 chromatin immunoprecipitation sequencing (ChIP-seq; GSE85631) and ERRγ binding sites defined by ERRγ ChIP-seq (GSE113784) in human induced pluripotent stem cell-derived cardiomyocytes (hiPSC-CMs). The heatmap in Fig. 3c was generated with the z-score presented in this table.

Supplementary Table 2. DNA oligonucleotide sequences for cloning

Cloning primer		
pcDNA3.1 (-)	Forward	Reverse
3xFLAG-ERRα	GCCCTCTAGACTCGAGCCACCATGGACTACAAAGACCATGACGGTGATTATAAAGATCATGACATCGATTACAAGGATGACGATGACAAGTCCAGCCAGGTGGTGGGCATTGAGC	GTGGCGGCCGCTCGATCAGTCCATCATGGCCTCG
3xFLAG-ERRγ	GCCCTCTAGACTCGAGCCACCATGGACTACAAAGACCATGACGGTGATTATAAAGATCATGACATCGATTACAAGGATGACGATGACAAGGATTCGGTAG	GTGGCGGCCGCTCGATCAGACCTTGGCCTCCAAC
GATA4-HA	GCCCTCTAGACTCGAGCCACCATGTATCAGAGCTTGGCCATG	GTGGCGGCCGCTCGATTAAGCGTAATCTGGAACATCGTATGGGTACGCAGTGATTATGTC
GATA6-HA	GCCCTCTAGACTCGAGCCACCATGGCCTTGACTGACGGCGG	GTGGCGGCCGCTCGATCAAGCGTAATCTGGAACATCGTATGGGTAGGCCAGGGCCAGGGC
pGL3-Basic	Forward	Reverse
TNNI3	CTAGCCCGGGCTCGAGCCTGGACTCTTGGGTCTGAG	GATCGCAGATCTCGAGGCGAGGTTCCCTAGCCTG
TNNI3 promoter vector for ERRE deletion	GCCTCCTGCCATTCCCGG	CAGACTAAATATACTGTCACCTCC
ERRE-deleted fragment	AGTATATTTAGTCTGCCTTTATCTCAGGTCTCAAGCAGC	GGAATGGCAGGAGGCGGGGCGTTTGGAGGGTCA
COX6A2	CTAGCCCGGGCTCGATTAAGGTACGAATTATTATACATTT	GATCGCAGATCTCGACAGCGCCAGCACGAAGGT
SMYD1	CTAGCCCGGGCTCGATCGAAGGTGTTTTGGGAGATTCA	GATCGCAGATCTCGAGGAATGAAAGCCCATTCAACTTT
pGL4.24	Forward	Reverse
MYH6-7 enhancer	GCTCGCTAGCCTCGATACCAGTGTGCTTAAAAACCCAGG	TCTTGATATCCTCGAGTAGGCAACAGATACACACATTATG
pAdTrack-CMV	Forward	Reverse
FLAG-ERRγ	GAAGGATCCGCCACCATGGACTACAAAGACGATGACGACAAGGATTCGGTAGAACTTTGC	CGGTAGAATTCTCAGACCTTGGCCTCCAACATTTC
Adeno-X CMV	Forward	Reverse
GATA4-HA	GTAACTATAACGGTCATGTATCAGAGCTTGGCCATGGCCGCCAACCAC	ATTACCTCTTTCTCCTTAAGCGTAATCTGGAACATCGTATGGGTACGCAGTGATTATGTC

Supplementary Table 3. DNA oligonucleotide sequences for mutagenesis

Mutagenesis	Forward	Reverse
GATA4 mutants		
pcDNA3.1 (-)-GATA4 S52F	GGGCCTGTTCTACCTCCAGGGCGGAGGC	AGGTAGAACAGGCCCAGCACGGAGG
pcDNA3.1 (-)-GATA4 P163S	GCCCCTACTCGGCTTACATGGCCGACG	AAGCCGAGTAGGGGCTGGAGTAGGAGC
pcDNA3.1 (-)-GATA4 E216D	GGCAGAGACTGTGTCAACTGTGGGGCTATGTC	GACACAGTCTCTGCCTTCTGAGAAGTCGTC
pcDNA3.1 (-)-GATA4 G296C	ATGCCTGCTGCCTCTACATGAAGCTCCACGG	AGAGGCAGCAGGCATTGCACACAGGC
pcDNA3.1 (-)-GATA4 G296S	ATGCCTGCAGCCTCTACATGAAGCTCCACGG	AGAGGCTGCAGGCATTGCACACAGGC
pcDNA3.1 (-)-GATA4 E395RfsX44	GCAGCAGCAGGAGATGCGTCCCATCAAGACG	ATCTCCTGCTGCTGCTGGTGGTGGC
pcDNA3.1 (-)-GATA4 L403M	TCTCGGCCATGAAGCTCTCCCCACAAGGC	GCTTCATGGCCGAGAGGACAGGGTGG
pcDNA3.1 (-)-GATA4 N-zinc Δ	GCAGAGAGGGCCTCTACCACAAGATGAACG	AGAGGCCCTCTCTGCCTTCTGAGAAGTCG
pcDNA3.1 (-)-GATA4 C-zinc Δ	GCCTCTCCGGCCTCTACATGAAGCTCCACG	AGAGGCCGGAGAGGCCCACTCGGCG
TNNI3-luc mutant	Forward	Reverse
pGL3-Basic-*TNNI3* promoter GATA binding sites deletion	GATCCAGCTGGGACCCTGAAGGTCACC	GGTCCCAGCTGGATCACCAGCCACC
ERRγ-Gal4 mutant	Forward	Reverse
ERRγ L449A-F450A-Gal4	ACAAAGCAGCATTGGAAATGCTGGAGGCC	CCAATGCTGCTTTGTGCATGGGCACTTTGCC
ERRγ M453A-L454A-Gal4	TGGAAGCAGCAGAGGCCAAGGTCTGACGTC	CCTCTGCTGCTTCCAAAAAAAGTTTGTGCATGGGC

Supplementary Table 4. DNA oligonucleotide sequences for qPCR and ChIP-qPCR

qPCR	Forward	Reverse
ACADM	TCCAGATCCTAAAGCTCCTGC	ACATCGCTGGCCCATGTTTA
ACSL1	ACTTGGGAAGGATTCTGGTCTG	GGCCTTTTCGCCTTCATTGT
ACSL3	TGGAGAGTTTGAACCCGATGG	AGAAACATATTCCCCTGCCTGT
ACTG1	CCGAGCCGTGTTTCCTTCC	GCCATGCTCAATGGGGTACT
ANKRD1	CGTGGAGGAAACCTGGATGTT	GTGCTGAGCAACTTATCTCGG
ATP1A3	TGACCTGCGGATCATCTCAG	GAGTCTGGGGCTCGGATTC
ATP5B	TGGCCACTGACATGGGTACTA	GGGTCAGTCAAGTCATCAGCA
BCL6	GTTGTGGACACTTGCCGGAA	CTCTTCACGAGGAGGCTTGAT
CKMT2	AGAAAGTGTGTGCCGAGGTC	ATGCAGTTGTTGTGCTTGCG
COX6A2	TTCCGTCCCTACCAACACCT	GGGTTCACGTGGCTATTGTG
CPT1B	GTGAGTGACTGGTGGGAAGAGTA	TGCACGTCTGTATTCTTGATGAG
CSRP3	GTGCCATCTGTGGGAAGAGT	TGTGTAAGGCCTCCAAACCC
ESRRA	AGCGAGAGGAGTATGTTCTAC	AGCCTCGGCATCTTC
ESRRB	TGCGAGATCACCAAACGGAG	GCATCCCCACTTTGAGGCAT
ESRRG	TTGGTGGCTGAACCGGA	CACACAGTGTAGTGAGGGCT
FABP3	CACAGCACCTTCAAGAACACA	AGTTTCCCTCCATCCAGTGTC
GATA4	GTG TCC CAG ACG TTC TCA GTC	GGG AGA CGC ATA GCC TTG T
GATA6	CTGCGGGCTCTACAGCAAG	GTTGGCACAGGACAATCCAAG
KCNQ1	ACCATCGGGCCACCATTAAG	CGCGCTTGCTGGAATTTCTT
LMOD2	TCATGGAAGCAATTCGGGGA	TTATCGCAGGGCTTCTGGAAC
MDH1	TGCTGTCATCAAGGCTCGAA	CTCCCTCTGGGGTTCCAAAC
MEF2C	GACCTCACGTCTGGTGCAG	TGCTTGCATATTCTTGTTCAAGTT
MYBPC3	GGTGGAGTTTGAGTGTGAAGTA	TGAACCGGTATTTGAAGGTCTC
MYH7	CTGTCCAAGTTCCGCAAGGT	ATTCAAGCCCTTCGTGCCAA
MYL3	GCCAAGACAGGAAGAGCTCAAT	CCTCATAGGTGCCTGTGTCC
PDK4	GGAGCATTTCTCGCGCTACA	ACAGGCAATTCTTGTCGCAAA
PLN	GACAGAGTCAGAAAACTCCCCA	AAAAGCTGGCAGCCAAATATGAG
PPARGC1A	AGTGACCAATCAGAAATAATATCCAATCAG	TGAGGACTGCTAGCAAGTTTG
PPARGC1A variant 8	TCTGGGTGGACTCAAGTGGT	TCCAAAGGATGTCCTTACCTCAAA
PPARGC1A variants 1-7	AGAGGCAGAAGGCAATTGAAGA	GTCCGTGTTGTGTCAGGTCT
PPARGC1B	CAGCCACTCGAAGGAACTTCA	CGGATGCTTGGCGTTCTG
RPLP0	TCTACAACCCTGAAGTGCTTGAT	GATAGAATGGGGTACTGATGCAA
RYR2	AGCCCTGAAGAATCGGCAAA	GACGTGCAAACGGTCTATGC
TNNC1	TGGTTCGGTGCATGAAGGAC	GTCGATGTAGCCATCAGCATT
TNNI1	CTCCTCTGGAACTGGGATTA	GCCTCATGGGTGGATAGA
TNNI3	GTGAAGAAGGAGGACACCGAG	CAAACTTTTTCTTGCGGCCC
TTN	CCCCATCGCCCATAAGACAC	CCACGTAGCCCTCTTGCTTC
ChIP-qPCR	Forward	Reverse
ATP5B	CGGCTCCAGGCATCCTTTTAACACCAC	CGGCGGTCCATCCTGGTAAGTGCTTTT
CPT1B	GTTTCTGTGCGGTGAAGGTT	CATCGGTGACCTTTTCCCTA
FABP3	TCTTGACCTTGCCTTGGGAC	GTCTGACCTTGTCCTGCAGT
MYBPC3	CTCTCTGCGTCCCTGACCT	TTGATTCTCGGTTTCTTTTTCC
POU5F1	CCCCAGGACAGAACCATCAC	GGTGTGGAGATTCCAGCCAA
TNNC1	AGGAGTGAGGGAGGCTGAG	CTATCCTGGGTGGTGGTGAC
TNNI3	CGTTTGGAGGGTCAGTGAG	CTGTGTCCTCGCCCTTTATC
MYH6-7 enhancer	CTCTAGTGCACCCCTTCACC	ATCTGCTAACCACGGTGTCC
NPPA-NPPB enhancer	CTTGGTGACATCTGGGCTTT	CTCACCACTGTTGGACGAAG

Supplementary Table 5. DNA oligonucleotide sequences for guide RNA

plentiCRISPRv2	Forward	Reverse
gRNA targeting rodent *Esrra*	CACCGCAGACCAGGCAGAGGCGTTT	AAACAAACGCCTCTGCCTGGTCTGC
gRNA targeting rodent *Esrrg*	CACCGGTGGGTCTTCCGACGCCAGT	AAACACTGGCGTCGGAAGACCCACC
Non targeting control (NTC)	CACCGATTGTTCGACCGTCTACGGG	AAACCCCGTAGACGGTCGAACAATC
pLV hU6-sgRNA hUbC-dCas9-KRAB-T2a-Puro	Forward	Reverse
gRNA targeting human *PPARGC1A*	CACCGGTGTGTGCTCTGTGTCACTG	AAACCAGTGACACAGAGCACACACC
