# Supplementary material for: The nuclear receptor ERR cooperates with the cardiogenic factor GATA4 to orchestrate cardiomyocyte maturation
Source: Nat Commun. 2022 Apr 13;13:1991. doi: 10.1038/s41467-022-29733-3 (PMC9008061; doi:10.1038/s41467-022-29733-3)
Supplement: Supplementary file 3 — Description of Additional Supplementary Files [file 41467_2022_29733_MOESM3_ESM.pdf]

Description of Additional Supplementary Files

File Name: Supplementary Data 1
Description: **Overlapped peak regions of cardiac super-enhancers and ERRγ binding sites in hiPSC-CMs**
The table shows the overlapped genomic regions of both cardiac super-enhancer regions defined by MED1 chromatin immunoprecipitation sequencing (ChIP-seq; GSE85631) and ERRγ binding sites defined by ERRγ ChIP-seq (GSE113784) in human induced pluripotent stem cell-derived cardiomyocytes (hiPSC-CMs).

File Name: Supplementary Data 2
Description: **Overlapped peak regions of ERRγ and GATA4 binding sites defined by the GATA4 and ERRγ ChIP-seq in hiPSC-CMs**
The table shows ERRγ and GATA4-overlapped peaks determined by the published GATA4 (GSE85631) and ERRγ (GSE113784) chromatin immunoprecipitation sequencing (ChIP-seq) information in human induced pluripotent stem cell-derived cardiomyocytes (hiPSC-CMs).
