# Supplementary material for: The nuclear receptor ERR cooperates with the cardiogenic factor GATA4 to orchestrate cardiomyocyte maturation
Source: Nat Commun. 2022 Apr 13;13:1991. doi: 10.1038/s41467-022-29733-3 (PMC9008061; doi:10.1038/s41467-022-29733-3)
Supplement: Supplementary file 6 — Reporting Summary [file 41467_2022_29733_MOESM6_ESM.pdf]

nature research

Corresponding author(s): Daniel P. Kelly

Last updated by author(s): Feb 14, 2022

Reporting Summary

Nature Research wishes to improve the reproducibility of the work that we publish. This form provides structure for consistency and transparency in reporting. For further information on Nature Research policies, see our Editorial Policies and the Editorial Policy Checklist.

Statistics

For all statistical analyses, confirm that the following items are present in the figure legend, table legend, main text, or Methods section.

n/a	Confirmed	
[ ]	[x]	The exact sample size (*n*) for each experimental group/condition, given as a discrete number and unit of measurement
[ ]	[x]	A statement on whether measurements were taken from distinct samples or whether the same sample was measured repeatedly
[ ]	[x]	The statistical test(s) used AND whether they are one- or two-sided<br>*Only common tests should be described solely by name; describe more complex techniques in the Methods section.*
[ ]	[x]	A description of all covariates tested
[x]	[ ]	A description of any assumptions or corrections, such as tests of normality and adjustment for multiple comparisons
[ ]	[x]	A full description of the statistical parameters including central tendency (e.g. means) or other basic estimates (e.g. regression coefficient) AND variation (e.g. standard deviation) or associated estimates of uncertainty (e.g. confidence intervals)
[ ]	[x]	For null hypothesis testing, the test statistic (e.g. *F*, *t*, *r*) with confidence intervals, effect sizes, degrees of freedom and *P* value noted<br>*Give P values as exact values whenever suitable.*
[x]	[ ]	For Bayesian analysis, information on the choice of priors and Markov chain Monte Carlo settings
[x]	[ ]	For hierarchical and complex designs, identification of the appropriate level for tests and full reporting of outcomes
[x]	[ ]	Estimates of effect sizes (e.g. Cohen's *d*, Pearson's *r*), indicating how they were calculated

Our web collection on statistics for biologists contains articles on many of the points above.

Software and code

Policy information about availability of computer code

Data collection	qPCR: QuantStudio Real-Time PCR Software v1.7<br>Western blotting:LICOR Image Studio Version 5.2.<br>Luciferase assay: BioTek Gen5 3.03<br>RNA-seq: MORPHEUS (https://software.broadinstitute.org/morpheus/), g:Profile (https://biit.cs.ut.ee/gprofiler/gost), Salmon 1.3.0, DEseq2 1.30.1.<br>ChIP-seq and ATAC-seq:Integrative Genomic Viewer 2.4.18, Bowtie2 2.4.1, Homer v4.11.1, g:Profile (https://biit.cs.ut.ee/gprofiler/gost), Toolkit for Cistrome DataBase Browser (http://dbtoolkit.cistrome.org/).<br>JASPAR (https://jaspar.genereg.net/).
Data analysis	RNA-seq: MORPHEUS (https://software.broadinstitute.org/morpheus/), g:Profiler May 2021 update (https://biit.cs.ut.ee/gprofiler/gost), Salmon 1.3.0, DEseq2 1.30.1.<br>ChIP-seq and ATAC-seq:Integrative Genomic Viewer 2.4.18, Bowtie2 2.4.1, Homer v4.11.1, g:Profiler May 2021 update (https://biit.cs.ut.ee/gprofiler/gost), Toolkit for Cistrome DataBase Browser (http://dbtoolkit.cistrome.org/), Python idr package v2.0.3 (https://github.com/nboley/idr), samtools 1.11.<br>JASPAR (https://jaspar.genereg.net/)<br>Microsoft Excel version 16 and Graph Pad Prism 8 or 9<br>R 4.1.2<br>Custom software for plotting the figures (https://github.com/batmanovkn/err_gata4_cardiomyocytes)

For manuscripts utilizing custom algorithms or software that are central to the research but not yet described in published literature, software must be made available to editors and reviewers. We strongly encourage code deposition in a community repository (e.g. GitHub). See the Nature Research guidelines for submitting code & software for further information.

Data

Policy information about availability of data

All manuscripts must include a data availability statement. This statement should provide the following information, where applicable:

- Accession codes, unique identifiers, or web links for publicly available datasets
- A list of figures that have associated raw data
- A description of any restrictions on data availability

GRCh38 (http://ftp.ensembl.org/pub/release-99/fasta/homo_sapiens/) was used as reference genome. ENSEMBL gene annotations v99 were used (http://ftp.ensembl.org/pub/release-99/gtf/homo_sapiens/). The RNA-seq, ChIP-seq, and ATAC-seq data generated in this study have been deposited in NCBI's Gene Expression Omnibus (GEO) under series accession number GSE166064 [https://www.ncbi.nlm.nih.gov/geo/query/acc.cgi?&acc=GSE166064]. ERRγ ChIP-seq data was available in NCBI's GEO with GSE113784 [https://www.ncbi.nlm.nih.gov/geo/query/acc.cgi?acc=GSE113784]. GATA4 and MED1 ChIP-seq data that support the findings of this study are available in NCBI's GEO with GSE85631 [https://www.ncbi.nlm.nih.gov/geo/query/acc.cgi?acc=GSE85631]. GATA6 ChIP-seq information was obtained from https://elifesciences.org/articles/53278/figures#content. The HFrEF RNA-seq data was obtained from online data repository (https://zenodo.org/record/4114617#.YWrnTNnMJ0w). Significantly regulated genes in G296S GATA4 hiPSC-CMs were obtained in https://doi.org/10.1016/j.cell.2016.11.033. Source data are provided with this paper. Uncropped immunoblot images are provided in Source Data file.

Field-specific reporting

Please select the one below that is the best fit for your research. If you are not sure, read the appropriate sections before making your selection.

[x] Life sciences [ ] Behavioural & social sciences [ ] Ecological, evolutionary & environmental sciences

For a reference copy of the document with all sections, see nature.com/documents/nr-reporting-summary-flat.pdf

Life sciences study design

All studies must disclose on these points even when the disclosure is negative.

Sample size	Power calculations were not performed prior to initiation of the study to determine sample sizes given we have done similar studies in past. Accordingly, sample sizes for quantification of gene and protein expression and luciferase assay were determined to obtain significance and reproducibility based on our previous experience and publications (https://doi.org/10.1161/CIRCRESAHA.119.316100 and https://doi.org/10.1161/CIRCRESAHA.114.302562). The sample sizes for ChIP-seq and ATAC-seq were also determined by the previous work (https://doi.org/10.1161/CIRCRESAHA.119.316100). 17588 open chromatin sites were defined by ATAC-seq, 68.2% of which contained H3K27ac. This result was similar to the recent publication in this field (https://doi.org/10.1016/j.cell.2016.11.033). Exact sample sizes are described in the manuscript.
Data exclusions	The following results were defined as an outlier with the ROUT (Q = 1%) method.<br>-Figure 3d, two NPPB results in WT Control.<br>-Supplementary Figure 6g, one ERRγ ChIP-qPCR result on MYH6-7 enhancer in siGATA4 #2 group.
Replication	The results were obtained from at least two or three independent experiments. All attempts at replication were successful.
Randomization	Randomization was applied when applicable. Cultured cells were allocated into experimental groups in random in all experiments. When processing ChIP samples, the samples were treated in random order.
Blinding	Sequencing on RNA-seq, ChIP-seq, or ATAC-seq library was blindly performed by GENEWIZ or the University of Pennsylvania Next-Generation Sequencing Core. The data was analyzed based on unbiased clustering for RNA-seq, ChIP-seq, and ATAC-seq analysis. Other experiments did not require blinding as the outcomes and findings were not influenced by the investigators' judgment and knowledge.

Reporting for specific materials, systems and methods

We require information from authors about some types of materials, experimental systems and methods used in many studies. Here, indicate whether each material, system or method listed is relevant to your study. If you are not sure if a list item applies to your research, read the appropriate section before selecting a response.

Materials & experimental systems

n/a	Involved in the study	
[ ]	[x]	Antibodies
[ ]	[x]	Eukaryotic cell lines
[x]	[ ]	Palaeontology and archaeology
[x]	[ ]	Animals and other organisms
[x]	[ ]	Human research participants
[x]	[ ]	Clinical data
[x]	[ ]	Dual use research of concern

Methods

n/a	Involved in the study	
[ ]	[x]	ChIP-seq
[x]	[ ]	Flow cytometry
[x]	[ ]	MRI-based neuroimaging

Antibodies

Antibodies used	Primary antibodies for western blotting<br>ERRα, SMYD1, VDAC, ACSL3, and TNNI3 (Abcam catalog and clone #s: ab76228; EPR46Y, ab181372; EPR13574(B)-30, ab15895, ab151959, and ab47003 respectively).<br>His tag, α-tubulin, and ACSL1 (Cell Signaling Technology catalog and clone #s: 12698S; D3I1O, 3873; DM1A, and 9189; D2H5 respectively).<br>HA tag and MYL2 (Proteintech, catalog #s 51064-2-AP and 10906-1-AP).<br>α-Actinin, FLAG M2, TNNI1, ACTB, and PGC-1α (Sigma-Aldrich catalog and clone #s: A7732-100UL; EA-53, F1804-50UG; M2, AV42117-100UL, A5316; AC-74, and ST1202; 4C1.3).<br>GATA4 and MYBPC3 (SANTA CRUZ BIOTECHNOLOGY catalog and clone #s, sc-25310; G-4, and sc-137180; E-7).<br>ERRγ (originally raised by Dr. Ronald Evans, and provided by Dr. Liming Pei). Purified specific ERRγ polyclonal antibody raised against the ligand-binding domain (amino acids 229–458); https://www.sciencedirect.com/science/article/pii/S155041310700068X?via%3Dihub#app2.<br>ERRγ (generated by the collaboration of Drs. Daniel Kelly and Anastasia Kralli). The Daniel Kelly and Anastasia Kralli laboratories designed a peptide (AcSNKDRHIDSSC-amide) that was used to raise the ERRγ antibody in a rabbit. The production of the antibody was completed by New England Peptide and was affinity purified.<br><br>All primary antibodies were diluted 1:1,000 except for anti-GATA4 and MYBPC3 (1:200).<br><br>Primary antibodies for ChIP and IP<br>H3K27ac (Abcam, ab4729).<br>ERRγ (generated by the collaboration of Drs. Daniel Kelly and Anastasia Kralli).<br>anti-HA-conjugated magnetic beads (Thermo Scientific, 88836).<br>Anti-DYKDDDDK Magnetic Agarose (Thermo Scientific, A36797).<br>Anti-c-Myc Magnetic Beads (Thermo Scientific, 88842).<br>mouse control IgG (Proteintech, B900620); 1 µg IgG was used to generate IgG-conjugated Dynabeads (Thermo Scientific, 10004-D).<br><br>Secondary antibodies for Western blotting<br>VeriBlot for IP Detection Reagent (Abcam, ab131366, 1:10,000 dilution).<br>IR Dye 800CW Donkey anti-Rabbit IgG (LICOR, 926-32213, 1:15,000 dilution).<br>IR Dye 800CW Donkey anti-Mouse IgG (LICOR, 926-32212, 1:15,000 dilution).<br>IR Dye 680RD Donkey anti-Mouse IgG (LICOR, 926-68072, 1:15,000 dilution).<br>IR Dye 680RD Donkey anti-Rabbit IgG (LICOR, 926-68073, 1:15,000 dilution).
Validation	ERRα: abcam, ab76228 (https://www.abcam.com/estrogen-related-receptor-alpha-antibody-epr46y-ab76228.html).<br>Abcam provides a western blotting image with ERRα KO HAP1 whole cell lysate. We also confirm the antibody specificity with our ERRα/γ KO hiPSC-CMs (https://www.ahajournals.org/doi/10.1161/CIRCRESAHA.119.316100).<br><br>SMYD1: abcam, ab181372 (https://www.abcam.com/smyd1-antibody-epr13574b-30-ab181372.html)<br>The protein expression was confirmed in WT Control hiPSC-CMs with this antibody, but it was not detected in non-cardiac cells (AD293) in Figure S4. SMYD1 is known to be expressed in myocytes.<br><br>VDAC: abcam, ab15895 (https://www.abcam.com/vdac1-porin-antibody-mitochondrial-loading-control-ab15895.html).<br>Abcam confirms that VDAC1 peptide blocks the antibody reaction.<br><br>ACSL3: abcam, ab151959 (https://www.abcam.com/acsl3-antibody-ab151959.html).<br><br>TNNI3:abcam, ab47003 (https://www.abcam.com/cardiac-troponin-i-antibody-ab47003.html)<br>In the publication (https://doi.org/10.1161/CIRCULATIONAHA.119.044205), this anti-TNNI3 detects the induction of TNNI3 protein expression in hiPSC-CMs during its maturation process.<br><br>His tag: Cell Signaling Technology, 12698S (https://www.cellsignal.com/products/primary-antibodies/his-tag-d3i1o-xp-rabbit-mab/12698)<br><br>α-tubulin: Cell Signaling Technology, 3873 (hhttps://www.cellsignal.com/products/primary-antibodies/a-tubulin-dm1a-mouse-mab/3873?_=1618844983952&Ntt=3873&tahead=true)<br><br>ACSL1: Cell Signaling Techng, 9189 (https://www.cellsignal.com//products/primary-antibodies/acsl1-d2h5rabbit-mab/9189)<br>In this publication (https://www.ahajournals.org/doi/10.1161/CIRCRESAHA.119.316100), the induction of protein expression during cardiac development is confirmed with this antibody. The expression is consistent with gene expression changes.<br><br>HA tag: Proteintech, 51064-2-AP (https://www.ptglab.com/products/HA-tag-Antibody-51064-2-AP.htm)<br><br>MYL2: Proteintech, 10906-1-AP (https://www.ptglab.com/products/MYL2-Antibody-10906-1-AP.htm)<br><br>α-Actinin: Sigma-Aldrich, A7732-100µl (https://www.sigmaaldrich.com/catalog/product/sigma/a7732?lang=en&region=US)<br>In Figure 1a, this antibody detected the induction of α-Actinin sarcomere protein expression during cardiac differentiation.

FLAG-M2: Sigma-Aldrich, F1804-50μl (https://www.sigmaaldrich.com/US/en/product/sigma/f1804?gclid=Cj0KCQjwpreJBhDvARIsAF1_BU3l3u9a-kiXRbe7xK9X2MnHQq_V-nyeNi8bFH0wRNXMTgO4PaXWNxQaAs2XEALw_wcB).

TNNI1: Sigma-Aldrich, AV42117-100μl (https://www.sigmaaldrich.com/catalog/product/sigma/av42117?lang=en&region=US)
This antibody is used in the previous publication (https://doi.org/10.1161/CIRCULATIONAHA.119.044205).

ACTB: Sigma-Aldrich, A5316 (https://www.sigmaaldrich.com/US/en/product/sigma/a5316?gclid=Cj0KCQjwpreJBhDvARIsAF1_BU1R_uP4znGctQUeVdD8wejd0_O1OZG3mmzGig2o-pVMeSAKHmWXnu4aAhLpEALw_wcB).

GATA4: SANTA CRUZ BIOTECHNOLOGY, sc-25310 (https://www.scbt.com/p/gata-4-antibody-g-4
gclid=Cj0KCQjw1PSDBhDbARIsAPeTqrcyHflCCGJN41W2aTMlDW9ufsqWK-2AMMPQYx2pBezbTAGDCqtvgkkaApXwEALw_wcB)
Knocking down GATA4 with siRNA decreased the bands which this antibody detected in Figure 5E.

MYBPC3: SANTA CRUZ BIOTECHNOLOGY, sc-137180 (https://www.scbt.com/ja/p/mybpc3-antibody-e-7)
In this publication (https://www.ahajournals.org/doi/10.1161/CIRCRESAHA.119.316100), MYBPC3 protein expression detected with this anti-MYBPC3 is found in only cardiomyocyte fraction but not non-cardiac fraction.

PGC-1α: Sigma-Aldrich, 4C1.3, ST1202 (https://www.sigmaaldrich.com/catalog/product/mm/st1202?lang=en&region=US)
Using PGC-1α knockout mouse, this antibody is validated by Sigma.

ERRγ: Dr. Liming Pei
In our and other publications (https://www.ahajournals.org/doi/10.1161/CIRCRESAHA.119.316100 and https://dx.doi.org/10.1128%2FMCB.01156-14), this antibody detects ERRγ protein expression in wild type mouse hearts, but not in ERRγ knockout hearts.

ERRγ: Dr. Anastasia Kralli
In our previous publication (https://www.ahajournals.org/doi/10.1161/CIRCRESAHA.119.316100), using ERRγ knockout hiPSC-CMs, ChIP peaks detected with this anti-ERRγ are defined as ERRγ specific peaks.

H3K27ac: Abcam, ab4729
Many publications including (https://doi.org/10.1038/nature22819) use this antibody for H3K27ac ChIP-seq.

anti-HA-conjugated magnetic beads: Thermo Scientific, 88836 (https://www.thermofisher.com/order/catalog/product/88836?ICID=search-88836#/88836?ICID=search-88836)
This bead is used for ChIP-seq in the publication (http://www.genesdev.org/cgi/doi/10.1101/gad.345686.120).

Eukaryotic cell lines

Policy information about cell lines

Cell line source(s)	α-Skin hiPSC: Provided by Dr. Huei-Sheng Vincent Chen (Indiana University School of Medicine)<br>WTC11 hiPSC: Provided by Dr. Deepak Srivastava (Gladstone Institutes)<br>AD-293:Agilent, 240085<br>293FT: ThermoFisher, R70007<br>H9C2:ATCC, ATCC® CRL-1446™<br>AC16: Sigma-Aldrich, SCC109
Authentication	We did not further authenticate the lines given that this has been done by the original creators of the lines.
Mycoplasma contamination	Mycoplasma test was routinely performed, and we confirmed that mycoplasma was not detected.
Commonly misidentified lines<br>(See ICLAC register)	No commonly misidentified cell lines were used.

ChIP-seq

Data deposition

☒ Confirm that both raw and final processed data have been deposited in a public database such as GEO.

☒ Confirm that you have deposited or provided access to graph files (e.g. BED files) for the called peaks.

Data access links<br>*May remain private before publication.*	All ChIP-seq data generated in this article have been deposited in NCBI's Gene Expression Omnibus Series accession number GSE166064.
Files in database submission	GSM5059557 H3K27ac ChIP-Seq in WT1<br>GSM5059558 H3K27ac ChIP-Seq in WT2<br>GSM5059559 H3K27ac ChIP-Seq in ERRγ KO1<br>GSM5059560 H3K27ac ChIP-Seq in ERRγ KO2

GSM5059561	Input in WT1 (ChIP-Seq)
GSM5059562	Input in WT2 (ChIP-Seq)
GSM5059563	Input in ERRγ KO1 (ChIP-Seq)
GSM5059564	Input in ERRγ KO2 (ChIP-Seq)

Genome browser session (e.g. UCSC)

Not available.

Methodology

Replicates

Two biological replicates.

Sequencing depth

All ChIP-Seq sequencing data has single end reads of length 51 base pairs.
Read depth:

Sample	raw reads	uniquely aligned reads
H3K27Ac ERRγ KO1	27358417	21490935
H3K27Ac ERRγ KO2	24325597	20041342
H3K27Ac WT1	27600174	21786308
H3K27Ac WT2	27834371	20600587
Input ERRγ KO1	29972034	26761011
Input ERRγ KO2	27874556	24708735
Input WT1	34411646	29105238
Input WT2	29087332	25399504

Antibodies

H3K27ac: Abcam, ab4729
Many publications including (https://doi.org/10.1038/nature22819) use this antibody for H3K27ac ChIP-seq.

Peak calling parameters

Alignment command line:

```
bowtie2 -p 50 -N 1 -x <index file> -U <fastq file> | samtools view -bSF4 - > <bam file1>
samtools sort -@ 16 -o <bam file 2> -O bam -T <bam file 2> -m 2G <bam file1>
samtools rmdup -s <bam file 2> <bam file 3>
samtools view -bq 1 <bam file 3> > <final bam file>
```

Index file is made from GRCh38 reference genome.

Peak calling with Homer:

```
makeTagDirectory <tag dir> hg38 -single <bam file>
findPeaks <target tag dir> -o <peak file> -i <input tag dir> -style histone
```

Peaks were called for WT and ERRgamma KO using the Input experiment as control.

Data quality

In WT, 28,602 H3K27ac peaks were identified with $p < 0.0001$ and > 4 fold signal over Input.
In KO, 31,110 peaks were identified with the same criteria. We checked the correlation between biological duplicate in H3K27ac ChIP-seq with each WT and ERRγ KO hiPSC-CMs. The duplicate in each genotype is highly correlated (WT r>0.99, ERRγ KO r=1.00). In addition, we have confirmed that the average H3K27ac ChIP-seq signals around transcriptional start sites of all genes has the expected shape in both WT and ERRγ KO hiPSC-CMs; the aggregation plots are provided as Supplementary Figure 3c.
To ensure data quality, we also looked at technical indicators: number of reads, percent of aligned reads, number of peaks found. All these showed good sequencing quality and ChIP signal. We looked at the tracks in IGV and found that the H3K27ac-enriched regions are large and diffuse, as expected. We plotted average spatial profile of H3K27ac signal at ERRγ peaks (previously published as GSE113784), and found a typical histone mark wave pattern, caused by depletion of nucleosomes at TF binding sites.

Software

Integrative Genomic Viewer 2.4.18, Bowtie 2.4.1, Homer v4.11.1
